# From one to many: The within-host rise of viral variants

**DOI:** 10.1371/journal.ppat.1009811

**Published:** 2021-09-02

**Authors:** Pierre Bessière, Romain Volmer

**Affiliations:** Ecole Nationale Vétérinaire de Toulouse, Université de Toulouse, ENVT, INRAE, IHAP, UMR 1225, Toulouse, France; University of Iowa, UNITED STATES

Over the course of a viral infection, a tremendous number of virions are produced: up to 10^14^—more than there are stars in the Milky Way [1].

These viruses do not exist as a genetically identical population. Genetic changes are constantly occurring, especially for RNA viruses, creating a cloud of genetically related variants [2]. Viral evolution within the infected host is shaped by host and viral factors.

Repeatedly, in the history of viral diseases, new strains have emerged with selective advantages, such as drug resistance or enhanced transmissibility. For example, a few months after the Severe Acute Respiratory Syndrome Coronavirus 2 (SARS-CoV-2) emergence, variants with enhanced infectivity have spread worldwide [3]. At the origin of each of these emergences, at least one infected host has been the scene of a competition between the newly arisen variant and its progenitors.

Viral populations face bottlenecks (events reducing the population size, and, therefore, its genetic diversity) that can be divided into 2 categories: transmission bottlenecks and within-host bottlenecks, which we will discuss here [4]. We propose that, in order to emerge, a variant has to overcome the within-host bottlenecks by winning the competition against its progenitors, allowing it to be transmitted and selected.

## A competition between the newly formed variant and its progenitors

When a variant appears from one progenitor through de novo mutations, it likely represents a minority variant, which can cause an evolutionary issue: Progenitor and offspring are replicating in parallel and most commonly target the same cell population. For the emergence to be evolutionary successful, the newly formed variant must be transmitted to another individual. Although transmission bottlenecks are intriguingly complex, becoming one of the majority variants likely increases the transmission probability. If a variant does not reach sufficient proportions, its transmission could be jeopardized. Conversely, beyond a certain proportion threshold, a variant will have more chances of being transmitted. An emergence is thus a 2-step process: firstly, the acquisition of a selective advantage, and, secondly, the ability to outcompete the progenitors and be transmitted to other individuals.

**Fig 1:**
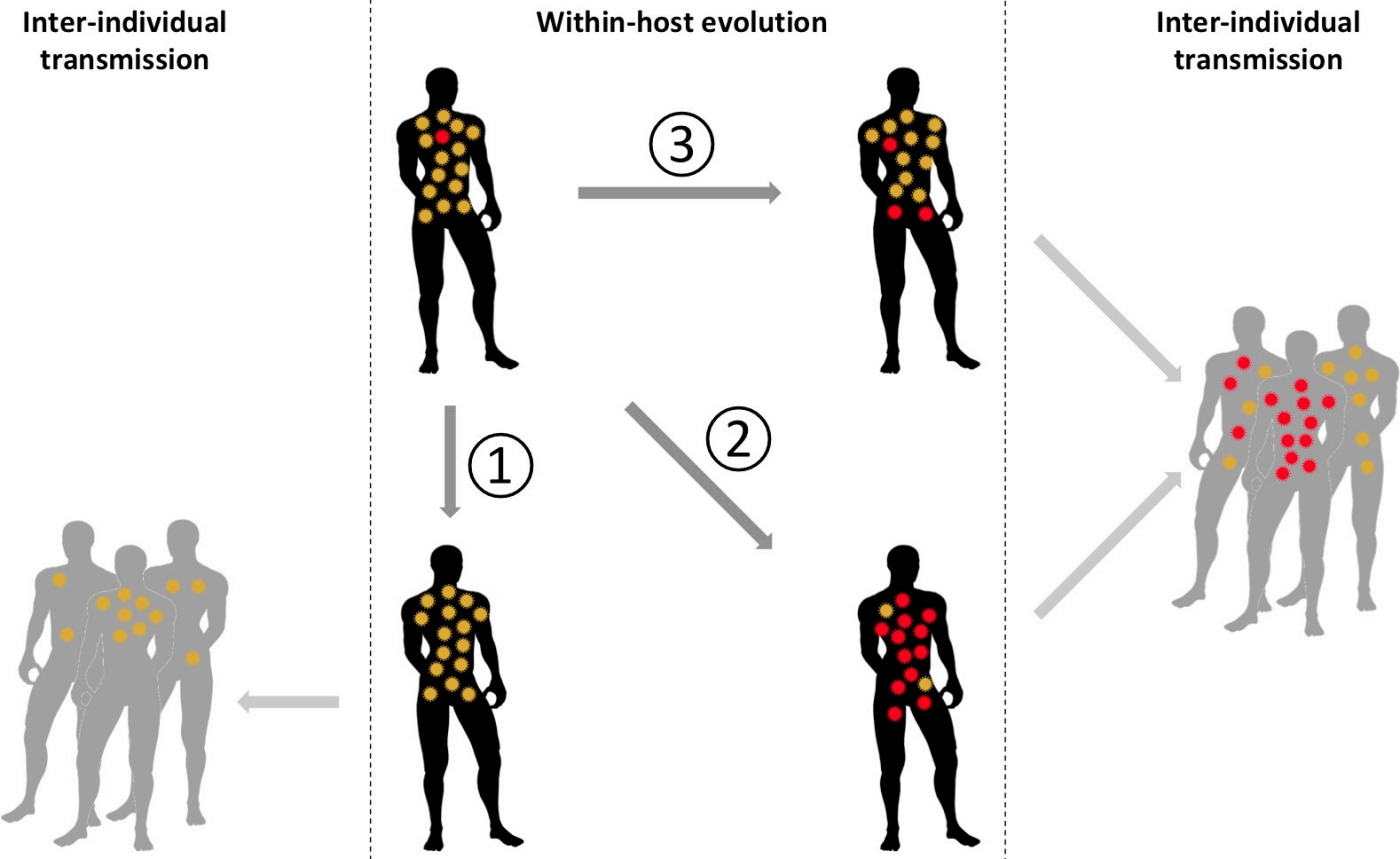
The 3 scenarios following variant apparition within one infected individual. Red: newly formed variant; yellow: progenitors.

In summary, we hypothesize that 3 scenarios can occur following the rise of a new variant within one individual (Fig 1). (1) The newly formed variant may fail to gain sufficient primacy over its precursors to be transmitted, resulting in a failed emergence. (2) The newly formed variant may outcompete or (3) coexist in lower proportions with its progenitors. In cases 2 and 3, the new variant may be transmitted, and the emergence can be evolutionarily successful.

Variants do not necessarily interact with each other in a competitive way: One variant might be able to provide a function missing in another variant, resulting in viral cooperation. Xue and colleagues elegantly demonstrated in vitro that coinfection with 2 influenza variants in different proportions, one variant being proficient at cell entry and the other at cell exit, converged to an equilibrium in which both variants reached approximately the same proportions [5]. However, synergistic interactions between variants of one viral species remain anecdotal compared to competitive ones [6].

## A huge selective advantage may not be enough

Intuitively, variants with a huge selective advantage, such as antiviral drug–resistant variants emerging during drug treatment or immune response–elusive variants, are likely to systematically gain primacy over their precursors. In practice, however, variants with substantial selective advantages may not always be selected, as it was demonstrated by coinfecting cells with different ratios of vesicular stomatitis virus (VSV) faster-replicating variants and wild-type virus [7]. Similarly, highly pathogenic avian influenza viruses, which reach higher viral loads and are more easily transmitted than their low pathogenic progenitors, are not always selected [8]. Thus, even if they have a selective advantage, variants may not always manage to become predominant enough and be transmitted if the within-host competition between the newly emerged variant and its progenitor is too strong. At least 3 major intertwined factors drive the within-host emergence of a minority variant: innate immune response, timing of appearance, and tissue specificity.

## Innate immunity prevents variants emergence

Rapidly, following infection, intracellular viral sensors trigger immune and inflammatory pathways, resulting in the establishment of an antiviral state, dominated by type I and type III interferons (IFNs) [9]. This immune response exerts a tremendous selective pressure on viral populations: By restraining viral replication, they increase the within-host competition. For example, in patients infected with IFN-sensitive strains of hepatitis C virus (HCV), type I IFN treatment resulted in a dramatic reduction in genetic diversity [10]. Also, by using influenza A–infected obese mice, which displayed a dampened innate immune response, a recent study showed that a delayed IFN response could lead to higher viral diversity and promoted minority variant emergence, while IFN treatment resulted in decreased viral diversity [11]. A strong type I IFN response may cause the disappearance of variants that arise. The innate immunity can therefore affect viral populations and prevent minority variants emergence through viral diversity reduction. However, under certain circumstances, innate immunity can also drive the emergence of less IFN-sensitive variants, as shown during HIV infection [12].

## Timing is critical

The acquisition of a mutation may occur at any time during the course of an infection. Logically, a long infection provides more time for de novo mutations to arise. Influenza viruses almost always gain oseltamivir resistance in immunocompromised oseltamivir-treated patients, who experience infections lasting weeks, if not months, while resistance does rarely arise in immunocompetent patients, for which viral clearance is usually a matter of days [13]. Logically, the earlier a new variant appears, the more likely it is to become predominant. If a variant was to appear in the very first viral replication cycles in a given host, the mutation(s) responsible for its selective advantage will be found at a higher frequency in the viral population [13]. On the contrary, a late-appearing variant will likely be a needle in a haystack, and the likelihood of its transmission to another host might be lower.

## Change in tissue tropism can promote variant emergence

Some mutations can affect tissue tropism. Notably, influenza A virus sialic acid receptors are not distributed equally along the respiratory tract. In humans, α2,6-linked sialic acids are mostly found in the upper respiratory tract, while the lower respiratory tract contains high proportions of α2,3-linked sialic acids [14]. Avian influenza strains preferentially bind to α2,3-linked sialic acids, and, consequently, avian influenza virus infections are predominantly restricted to lower lungs in humans [14]. If an avian-derived variant able to bind α2,6-linked sialic acids emerged within a human, it would have the opportunity to reach the upper respiratory tract and replicate remotely from its progenitors. As a consequence, we hypothesize that this variant would reach a niche where there is no competition with its progenitors for resources and where local immunity is still low. Change in tissue tropism is also well illustrated by other viruses, such as duck Tembusu virus (TMUV) or porcine respiratory coronavirus (PRCV). The P156S variant of TMUV, a mosquito-borne virus belonging to the Flaviridae family, is able to replicate efficiently in the respiratory tract, while its progenitor mostly replicate in the spleen [15]. Similarly, PRCV emerged following a deletion in the spike gene of its progenitor, the transmissible gastroenteritis virus (TGEV). While TGEV replicates in the digestive tract, PRCV replicates in the respiratory system [16]. These changes in tropism certainly promoted emergence through the acquired ability to be transmitted through respiratory droplets. However, successful emergence may have been further promoted by the lowered within-host competition of these variants with their progenitors.

## Concluding remarks

A variant may fail to be transmitted because it appeared too late and did not have time to reach sufficient proportions before the infection was abrogated by the host immune response. Similarly, when a viral infection results in the death of its host, the transmission likelihood diminishes with the time interval between variant emergence and host’s death. Thus, if a variant was to gain a huge selective advantage, without the right conditions, its emergence would come to nothing. These obstacles likely limit the emergence of potentially dangerous variants—for example, no mammalian to mammalian transmissible H5N1 virus has emerged yet, even though studies in the ferret model have suggested that only a few amino acid substitutions were required for airborne transmission [1].

At a population level, other factors can prevent transmission. A selective advantage in one context can be detrimental in another, depending on the host genetic background and immune status [17]. Vaccination provides specific immunity and limits transmission, but co-circulating viruses may also hamper variants emergence. Infection by one virus can prevent or at least delay infection with a different virus through type I and type III IFNs and the induction of a nonspecific antiviral state [18,19].

Deep sequencing technologies, mathematical modeling, and in vivo infections with mixtures containing variants at different ratios provide the tools for within-host viral diversity analysis and for a better understanding of the epidemiological and evolutionary forces that drive variants emergence.
